# Effect of superamphiphobic macrotextures on dynamics of viscous liquid droplets

**DOI:** 10.1038/s41598-018-33656-9

**Published:** 2018-10-18

**Authors:** Asif Raiyan, Tabor Scott Mclaughlin, Rama Kishore Annavarapu, Hossein Sojoudi

**Affiliations:** Department of Mechanical, Industrial and Manufacturing Engineering, The University of Toledo, Toledo, 43606 Ohio USA

## Abstract

The ability of hydrophobic surfaces to repel impinging liquid droplets is important in applications ranging from self-cleaning of solar panels to avoiding ice formation in freezing rain environments. In quest of maximizing water repellency, modification of droplet dynamics and subsequent reduction of contact time have been achieved by incorporating macrotexture on the superhydrophobic surfaces. However, the dynamics of low temperature water, and other viscous liquid droplets impacting anti-wetting surfaces with macrotextures is not well explored. Here, we investigate the effect of viscosity on the bouncing dynamics of liquid droplets impacting macrotextured superamphiphobic surfaces using various glycerol-water mixtures as model liquids at different impacting conditions. We demonstrate that the changes of reduction in contact times by macrotextures due to the increasing viscosity are in opposite trends at low and at high impact velocities. Since macrotexture executes substantial contact time reduction for the droplets which exhibit splitting after the impact, a preliminary model for predicting the minimum impact velocity to observe droplet splitting by macrotexture is proposed considering the important parameters of an impinging droplet along with the surface characteristics and the macrotexture size. This work aims to provide an insight on several possible outcomes of viscous droplets impacting on the macrotextured surfaces and a model that will help to design the desired superamphiphobic surfaces capable of exhibiting reduced contact time and enhanced repellency of low-temperature water droplets (such as freezing rain) and other viscous liquids (such as oils) under different impacting conditions.

## Introduction

Liquid droplets impacting onto solid surfaces is a common phenomena encountered in nature and is important for many industrial applications such as pesticide deposition, spray cooling of hot surfaces, spray painting and coating, ink-jet printing, microfabrication, and impact erosion^1–8^. The studying and understanding the bouncing dynamics of liquid droplets impacting on solid surfaces with special wetting properties help to design surfaces that execute self-cleaning, reduce erosion, increase the efficiency of condensers and steam turbines, guide or trap liquids, perform oil-water separation, and avoid or minimize the ice formation on solar photovoltaics, offshore oil platforms, wind turbines, aircrafts and other structures^6,9–18^. For the past two decades, droplet repellency on the hydrophobic and superhydrophobic solid surfaces has been an active research field^6,19–26^. Superhydrophobic surfaces (SHS) show excellent anti-wetting properties characterized by the higher water contact angles (WCA > 150°) and very small contact angle hysteresis (difference between advancing and receding contact angle)^27^. The superhydrophobic surface typically has very low surface energy with micro-scale, nano-scale, and/or hierarchical features which entrap a thin air pocket between the droplet and substrate^23,28–31^. The air pockets help to reduce the energy dissipation by decreasing the contact area between the droplet and the surface textures. Similarly, superoleophobic surfaces which have low surface energy and re-entrant features repel oil droplets. However, oils and other organic liquids usually exhibit higher surface attraction due to their lower surface tension compared with water. Therefore, it is more difficult to fabricate a superoleophobic surface than a superhydrophobic surface^32^. Surfaces showing repellency towards not only water but also low-surface-tension liquids such as oils are called superamphiphobic surfaces^33^.

When a liquid droplet impinges on an anti-wetting surface, there are four major possible outcomes: homogeneous wetting, splashing, bouncing off, and impalement/sticking^5,34,35^. Three important forces affecting these outcomes are inertial, viscous, and capillary forces. These forces are dependent on factors associated with the droplet (size, density, surface tension, impact velocity, viscosity, and temperature) and the surface (chemistry, roughness, and temperature). The states of wetting (impalement and bouncing) are dependent on the balance between wetting and anti-wetting pressures^34,36^. The wetting pressures are the hammer pressure and the dynamic pressure, whereas the anti-wetting pressure is the capillary pressure. At the first instant of droplet impact, the contact between the droplet and the textured surface generates a hammer pressure and during the spreading stage, the dynamic pressure is dominant. On the other hand, the capillary pressure is generated within the surface textures and impedes the impalement of the droplet into them. The impacting droplet does not fully rebound if the impact velocity (*v*) is higher than a certain value, which is known as critical velocity (*v*_c_)^37^. In such a case, wetting pressure is higher than the resistive capillary pressure and the impacting drop remains partially or entirely attached to the surface, which is known as an impalement or sticking phenomena^37^. If the impact velocity is below *v*_c_, the impacting droplet has contact only with the highest parts of the surface texture, so the air pockets remain trapped between the droplet and the substrate, avoiding the impalement^34^. Deng *et al*. have mentioned that this bouncing phenomena occurs when the capillary pressure is greater than wetting pressures, following a completely non-wetting state^36^. However, if a droplet strikes the surface with sufficient kinetic energy (e.g. *v* > *v*_c_), it may displace the trapped air pockets between the features and become pinned (Wenzel state)^15,34,38^.

For a bouncing droplet, the duration of time the droplet is in contact with the surface is called the contact time. When the droplet bounces off the surface, the amount of mass, momentum, and energy exchanged are significantly governed by this contact time^39^. Therefore, minimizing the contact time may be advantageous in certain applications like reducing heat transfer between a surface and impacting liquids^22,40^. It has previously been reported that the contact time of a bouncing water droplet is not dependent on the impact velocity over a wide range of Weber number (*We* = *ρv*^2^*R*_0_*/σ*, where *ρ*, *v*, *R*_0_ and *σ* are the liquid droplet density, impact velocity, initial droplet radius and surface tension of the liquid, respectively) though the details of the intermediate stage deformation of droplets significantly depend on it^21,41^. Richard *et al*. have mentioned that these droplet deformations are comparable to harmonic oscillation, although they show non-linear deformation, unlike harmonic oscillation^21^. The droplet contact time with the surface is proportional to the inertial-capillary time scale which is characterized by $\tau =\sqrt{(\frac{\rho {R}_{0}^{3}}{\sigma })}$ ^21,42^. However, for the higher range of Weber numbers, we noticed a significant reduction in contact time of water droplet due to splashing.

Researchers have been trying to maximize the liquid repellency by surfaces in which reducing the contact time is often beneficial for the purpose^22,39^. Bird *et al*. have demonstrated that the contact time is significantly affected by the splitting of the droplet using a macrotexture as a ridge on a flat superhydrophobic surface^22^. They mentioned that since dewetting can start from both the macrotexture and the edges of split drops, recoiling distance is divided by almost two, compared with regular rebounds. Thus, macrotexture alters the dynamics of a water droplet during the recoiling phase, reducing the contact time by around 40%. Later, Gauthier *et al*. have simplified this apparently-complex problem by applying the inertia-capillary nature of the water droplet^40^. They have shown that if a droplet is deformed in such a way that the droplet has *n* number of lobes, the contact time of the droplet on a macrotextured superhydrophobic surface becomes ${t}_{c}=\frac{{t}_{0}}{\sqrt{n}}$, where *t*_0_ is the contact time of the droplet on a surface without the macrotexture. They studied the reduction in droplet contact times with different impact velocities, droplet sizes, and macrotextures sizes. Along with these two fundamental works^22,40^, other studies have also shown reduction in contact time by incorporating one and/or multiple macrotextures^27,43,44^. Despite recent studies, fundamentals of viscous liquid droplet dynamics on macrotextured superamphiphobic surfaces is still unknown. For designing the macrotextured icephobic surfaces operating in freezing rain environments, viscous effect should be considered since viscosity of water droplets increases at low temperatures, which significantly changes the droplet contact time. In addition, maximizing oil repellency from surfaces using macrotexture is of great interest for applications in oil-water separation, oil flow and ink-jet printing. Nevertheless, no work has been reported which study the dynamics of viscous liquid droplets impacting superamphiphobic macrotextures for reducing the viscous dissipation and subsequently the droplet contact time. The previous explanations for contact time reduction was applicable only for water droplets and do not hold for viscous liquids. Here we focus on studying the viscous effects on the droplet dynamics with and without macrotexture, investigating the conditions for observing the splitting of liquid droplets with various viscosities. The impact velocity at which the droplet is split by the macrotexture is a very important factor, responsible for the significant reduction in contact time and the maximization of viscous liquid repellency. Unfortunately, not much attention has been given to the threshold impact velocity for non-splitting to splitting transition of the droplet in the previous works. Here, we introduce a threshold impact velocity, which is referred to as the minimum impacting velocity for observing droplet splitting (*v*_MS_). The complexity of viscous dissipation along the macrotexture and on the superamphiphobic surface is studied experimentally and then a new model is proposed for understanding when the macrotexture can induce maximum repellency of the viscous liquids on the superamphiphobic surfaces. A novel model for predicting the *v*_MS_ value is proposed considering important parameters of an impinging droplet along with surface characteristic parameters such as contact angles and macrotexture dimensions. In addition, we demonstrate that the critical impact velocity (*v*_c_) of a viscous liquid droplet impacting superamphiphobic surfaces are affected remarkably by the presence of the macrotexture. At the critical impact velocity, the droplet impales into the flat surface without the macrotexture whereas the droplet with the same impact velocity splits and bounces off the surface with macrotexture, avoiding the impalement. This study provides an insight on several possible outcomes of impacting viscous droplets on the macrotextured surfaces and a model that will help to design the desired superamphiphobic surfaces for many applications such as avoiding ice formation during freezing rain or guiding inks in inkjet printing.

## Results

### Superamphiphobic surface

**Figure 1:**
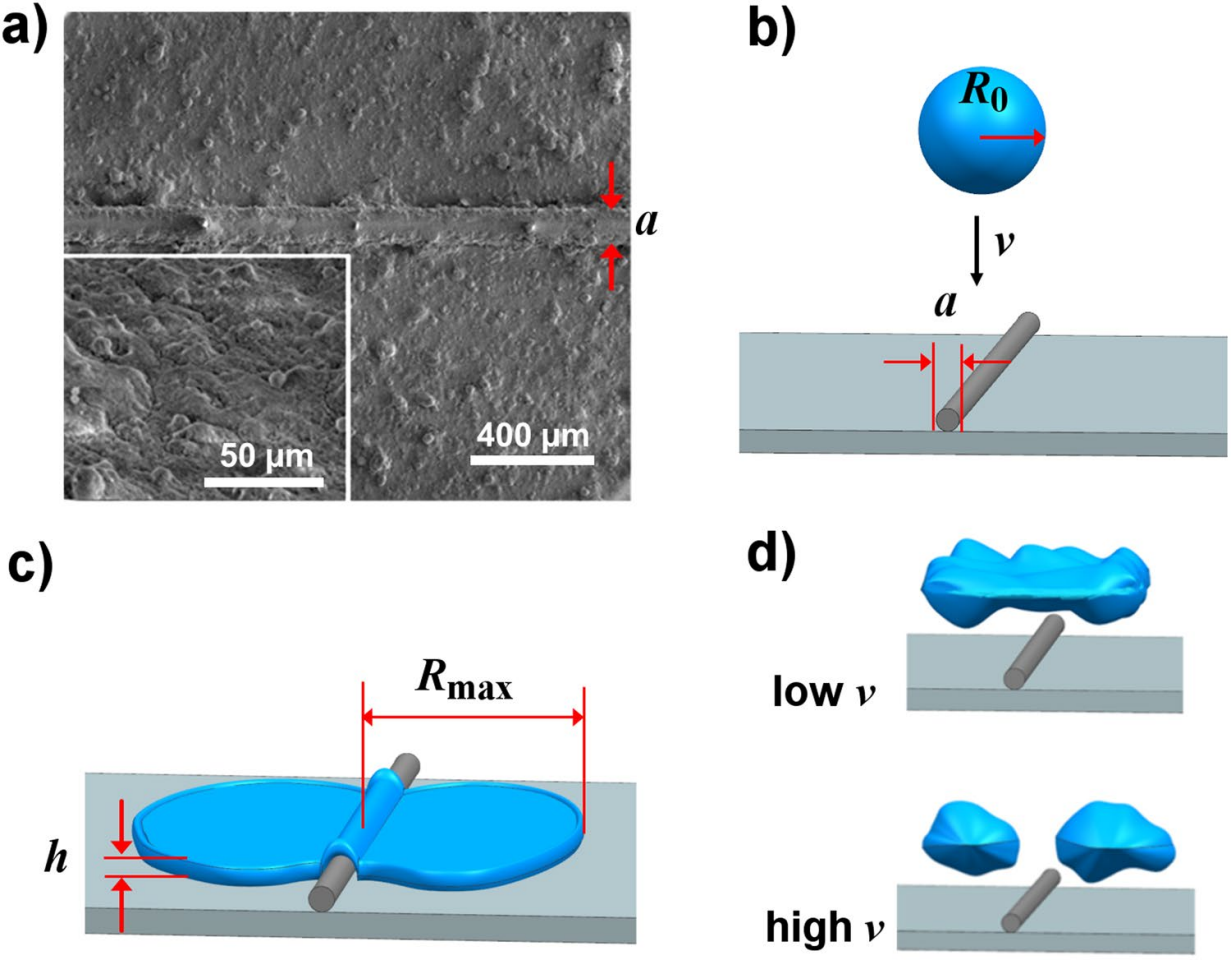
Material and schematics of droplet dynamics. (**a**) Scanning electron microscope images of the superamphiphobic coating deposited on a silicon substrate with a macrotexture of *a* = 150 µm diameter. Inset: close-up view of the texture, showing aggregated colloidal beads with typical sizes of 5–10 µm. (**b**) Schematic of a liquid droplet with radius *R*_0_, impinging on a macrotexture with diameter *a* on the surface with impact velocity of *v*. (**c**) Schematic of a liquid droplet after spreading, which shows maximum spreading radius of *R*_max_ and thickness of *h*. (**d**) Schematic of the two different outcomes of the impacting droplet on the macrotexture. Droplet exhibits non-splitting (top) and splitting (bottom) phenomena at a low and a high impact velocity (*v*), respectively.

A tinned-copper wire of diameter *a* = 150 µm is placed on a flat silicon substrate. Both the wire and the substrate are then coated with a commercial spray (Rust-Oleum® NeverWet® Liquid Repelling Treatment). The coating creates a macrotextured anti-wetting surface. The surface morphology of the superamphiphobic sample was examined by the images captured using scanning electron microscope (SEM). The SEM images show aggregates of colloidal beads whose typical sizes are 5–10 µm, both on the substrate and the wire (Fig. 1a**)**, making both similarly superhydrophobic. Flat superamphiphobic surfaces are made in the similar way, but without the wire.

To study the viscous effect, solutions with various concentrations of glycerol and water are made. Glycerol-water solutions of 0%, 40%, 60%, and 70% by weight are made to obtain mixtures with a wide range of viscosity value (*µ*) while maintaining close density (*ρ*) and surface tensions (*σ*) values as shown in Table 1. The changes in viscosity values are not significant for glycerol-water weight ratios 0–30% or 40–50%. Therefore, a larger range of glycerol-water ratios was selected to clearly distinguish the effects of viscosity on droplet bouncing and subsequent contact times.

**Table 1:** Density (*ρ*), surface tension (*σ*), dynamic viscosity (*µ*), static contact angle (*θ*_s_), advancing contact angle (*θ*_a_), and receding contact angle (*θ*_r_) for various glycerol-water mixture liquids.

Glycerol weight (%)	Density^55^ *ρ* (g/cm^3^)	Surface tension^56^ *σ* (mN/m)	Dynamic viscosity^55^ *µ* (mPa.s)	Static contact angle *θ*_s_ (°)	Advancing contact angle *θ*_a_ (°)	Receding contact angle *θ*_r_ (°)
0	1.00	72.0	1.0	162.3 ± 0.4	165.6 ± 1.7	157.8 ± 2.0
40	1.09	65.9	3.7	161.9 ± 1.7	164.2 ± 1.0	155.4 ± 1.7
60	1.15	64.6	10.8	161.2 ± 2.1	163.3 ± 1.2	155.0 ± 1.3
70	1.18	63.8	22.5	160.6 ± 1.0	162.0 ± 0.3	154.9 ± 2.3

Static contact angle (*θ*_s_), advancing contact angle (*θ*_a_), and receding contact angle (*θ*_r_) for these various glycerol-water mixture liquids are measured on the coated surface (Table 1). All gently deposited liquid droplets exhibited high static contact angles (>150°). Advancing contact angle (*θ*_a_) is always larger than or equal to the receding contact angle (*θ*_r_) and their difference is called the contact angle hysteresis. The water droplet (0% glycerol-water solution) exhibited a static contact angle (*θ*_s_) of 162.3° (an average of ten readings) on the prepared samples. Advancing and receding contact angles for water are *θ*_a_ = 165.6 ± 1.7° and *θ*_r_ = 157.8 ± 2.0°, respectively. The values of contact angle hysteresis are found to be very small (<10°) for the various glycerol-water mixture droplets on the coated surface (Table 1). From the high static contact angles (*θ*_s_) and low contact angle hysteresis (*θ*_a_ − *θ*_r_) values, we verified the sample as a superamphiphobic surface, suitable to perform our experiments with the viscous liquid droplets of radius *R*_0_ and impact velocity *v* (Fig. 1b). The schematics of maximum droplet spreading with radius of *R*_max_ and thickness of *h* are shown in Fig. 1c. Two different outcomes of the impacting droplets on the superamphiphobic macrotexture: non-split droplet at a low impact velocity (top) and split droplets at a high impact velocity (bottom) are shown in Fig. 1d.

### Effect of viscosity on flat superamphiphobic surface

**Figure 2:**
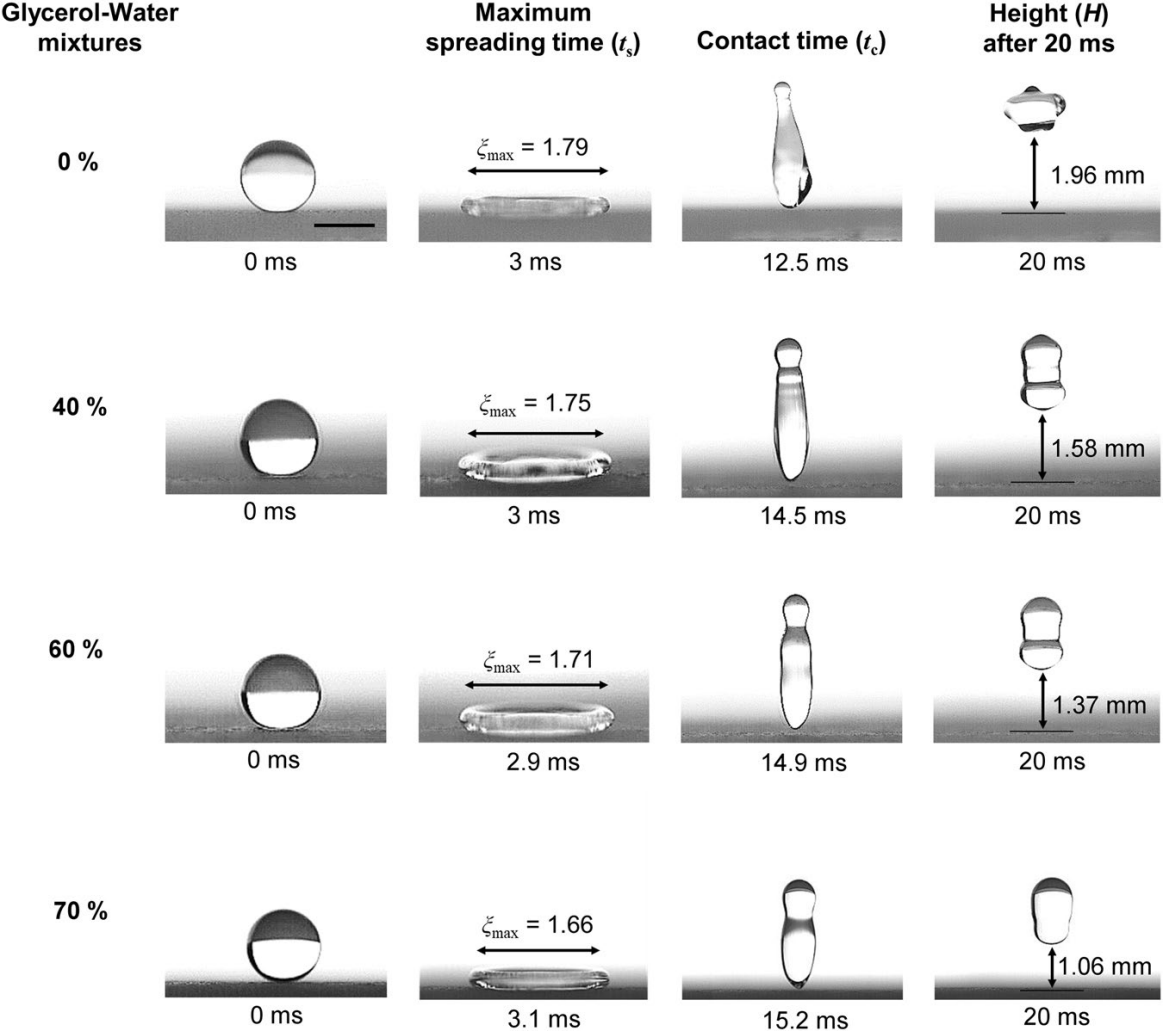
Effect of viscosity at impact velocity of 1 m/s (Weber number, *We* = 18~24). Side views of various glycerol-water mixture (0%, 40%, 60%, and 70% by weight) droplets (radius, *R*_0_ = 1.3 mm) impacting and rebounding from the superamphiphobic surface without a macrotexture. The spreading time (*t*_s_) is almost identical, although the maximum spreading factor (*ξ*_max_ = *R*_max_/*R*_0_) decreases with increasing viscosity (2^nd^ column) due to low spreading speed. The contact time (*t*_c_) increases for viscous liquid droplets (3^rd^ column) and the heights of the droplets (*H*) after a certain time (20 ms from impact) decreases with higher viscosity (4^th^ column) due to the higher energy dissipation. The scale bar represents 2 mm.

An impacting droplet on a superamphiphobic surface spreads and recoils over the trapped air pockets so quickly that it completely bounces off the surface. Different stages of the bouncing phenomena of various glycerol-water mixture (0%, 40%, 60% and 70% by weight) droplets are demonstrated in Fig. 2. The droplets with radius of *R*_0_ = 1.3 mm impinge on the flat superamphiphobic surface without macrotexture at an impacting velocity of *v* = 1 m/s (Weber number, *We* = 18~24). The columns from left (1^st^) to right (4^th^) represent the moments of the droplet just before impact, at maximum spreading condition, when leaving the surface, and after 20 ms of the impact, respectively. We define time *t* = 0 ms as the moment just before the droplet has contact with the surface (first column). Upon impact, the droplets spread until the surface tension and viscosity overcome the inertial forces, leading to their maximum spreading (2^nd^ column). The droplets demonstrated almost identical maximum spreading time (*t*_s_ ~ 3 ms) despite their considerable differences in viscosity. However, the maximum spreading radius (*R*_max_) decreases at higher viscosity. Maximum spreading factor (*ξ*_max_) is defined by the ratio of the maximum droplet spread radius (*R*_max_) and the initial radius of the spherical shape droplet (*R*_0_) before the impact. Maximum spreading factor (*ξ*_max_ = *R*_max_/*R*_0_) was found to be 1.79, 1.75, 1.71 and 1.66 for 0%, 40%, 60%, and 70% glycerol-water mixture droplets, respectively. The droplet contact time (*t*_c_) increases for liquid droplets with higher viscosity (3^rd^ column), being 12.5 ms, 14.5 ms, 14.9 ms and 15.2 ms for the increasing viscous droplets, respectively. From the observations of spreading times and spreading factors, we conclude that spreading speed is slower for liquid droplets with higher viscosity. In addition, from the retraction distance (2*R*_max_) and the retraction time (difference between contact time, *t*_c_ and spreading time, *t*_s_), it is evident that the retraction speed is slower for liquid droplets with higher viscosity. The heights of the droplets (*H*) at a certain time (20 ms) after impact are also shown in the Fig. 2 (4^th^ column). The values of *H* were found to be decreasing (1.96 mm, 1.58 mm, 1.37 mm, and 1.06 mm, respectively) with the increase in the viscosity.

**Figure 3:**
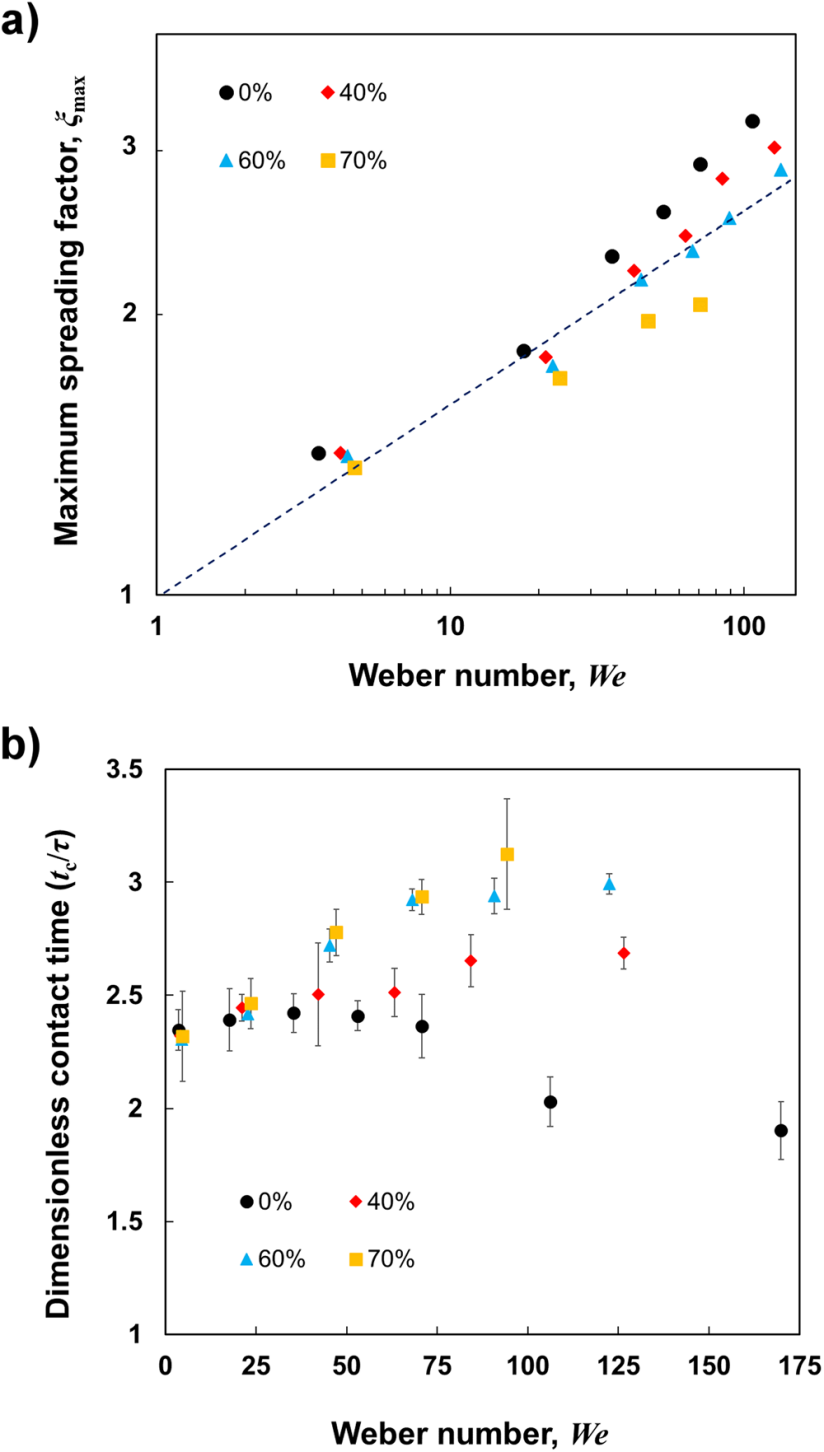
Maximum spreading and contact time of bouncing droplet. (**a**) Maximum spreading factor (*ξ*_max_ = *R*_max_/*R*_0_) as a function of Weber number, *We* for various glycerol-water mixture (0%, 40%, 60%, and 70% by weight) droplets after impacting on a surface without a macrotexture. The dashed line indicates the slope of ¼. For this range of viscosity, *ξ*_max_ correlates with $W{e}^{\frac{1}{4}}$, supporting the scaling of capillary-inertia dominated spreading. (**b**) Dimensionless contact time (*t*_c_/*τ*) as a function of Weber number, *We* for various glycerol-water mixture (0%, 40%, 60%, and 70% by weight) droplets after impacting on a surface without macrotexture. Contact times for water droplets (black circles) are almost identical for different Weber numbers (except for high Weber numbers where splashing occurs) whereas for liquid droplet with higher viscosity, they increase with Weber numbers. The contact times for viscous droplets at very high Weber numbers are not shown since the droplet did not rebound. Error bars represent the standard deviation of the experimental values.

Similar experiments were conducted with a wide range of impact velocity on this flat superamphiphobic surface. Maximum spreading factors (*ξ*_max_) were measured for droplets with different viscosities and are plotted as function of Weber numbers (*We*) (Fig. 3a). For viscous liquids on a superamphiphobic surface, the maximum spreading factor correlates closely with *We*^1/4^. This indicates that the spreading is determined by the capillarity and the inertia, and the viscous effect is small but not negligible as we can see in the next plot (Fig. 3b) more clearly^45,46^. The dimensionless contact times (*t*_c_/*τ*) of various glycerol-water mixture droplets as function of Weber numbers (*We*) are shown in Fig. 3b. Contact times of water droplets (black circle) for different impact velocities (or Weber numbers) are almost identical (*t*_c_/*τ* ~ 2.4 ms), except for high Weber numbers. This statement agrees with previous observations that suggested that the contact time of a bouncing water droplet scales with inertia-capillary timescale (*τ*)^21^. However, at a high impact velocity or Weber number (*We* > 100), the kinetic energy of the water droplet is dominant over capillary energy, leading to the splashing phenomena. During splashing, the water droplet is completely lifted off the surface, with some satellite droplets forming around the main droplet. The satellite droplets start leaving the main droplet as soon as spreading begins while the main droplet is the last one to bounce off and leave the surface. We consider only the contact time of the main droplet when splashing occurs. Here, reduction of contact time is experienced even without any macrotexture on the surface at high Weber number. On the contrary to the water droplets, contact time increases for the viscous liquid (40%, 60%, and 70% glycerol-water mixture) droplets with higher impact velocities (or Weber numbers) (Fig. 3b). However, the dimensionless contact times are almost identical for all liquid droplets at the very low Weber numbers.

The effect of viscosity can be understood more vigorously by the energy conversion of the impacting droplet. Before impacting the surface, the total energy of a droplet is comprised of kinetic energy and surface energy. The kinetic energy (*KE*_1_) and surface energy (*SE*_1_) of the spherical droplet before impacting the surface are given by the following relations:

1 $$K{E}_{1}=\frac{2}{3}\rho \pi {R}_{0}^{3}{v}^{2}$$

2 $$S{E}_{1}=4\pi \sigma {R}_{0}^{2}$$

When the droplet impacts the surface, the kinetic energy (inertial force) causes the droplet to spread. During the spreading, the kinetic energy is consumed and transformed into interfacial surface energy and dissipation energy^25^. Since the surface energy of a superamphiphobic surface is very low, energy dissipation due to friction is not significant, and can be neglected^47^. However, viscous energy dissipation during spreading and retraction must be considered. Impacting droplets spread on the surface until the droplet’s viscosity and surface tension overcome the inertial forces of the droplet. The viscous dissipation energy (*E*_diss_) can be estimated using the work done by the viscous friction force. From the original expression of Chandra and Avedisian^35^, the dissipation function (*Φ*) and dissipated energy (*E*_diss_) are given by the following relations:

3 $$\Phi \approx \mu {(\frac{v}{h})}^{2}$$

4 $${{E}}_{{\rm{diss}}}={W}={\int }_{0}^{{t}_{s}}{\int }_{{\Omega }}{\rm{\Phi }}\,d{\rm{\Omega }}\,{\rm{d}}{t}_{s}=\Phi \Omega {t}_{s}\approx \mu {(\frac{v}{h})}^{2}\Omega {t}_{s}$$

where *Ω* and *t*_s_ are the volume of a viscous liquid droplet and the time of spreading, respectively. Assuming the impinging droplet as a cylindrical shape at the maximum spreading (i.e. $\Omega \approx \pi {R}_{{\rm{\max }}}^{2}h$), the above equation becomes:

5 $${E}_{{\rm{diss}}}\approx \frac{\pi \mu {v}^{2}{R}_{{\rm{\max }}}^{2}{t}_{s}}{h}$$

The above energy dissipation (*E*_diss_) relation explains the combined effect of viscosity and velocity on bouncing liquid droplet. The dissipated energy during spreading is larger for the droplet with higher viscosity^48^, therefore the maximum spreading of a droplet with a certain kinetic energy changes inversely with viscosity as shown in Fig. 2 (second column).

At maximum spreading, the kinetic energy becomes zero and the interfacial surface energy (*SE*_2_) becomes $S{E}_{2}=\pi {R}_{0}^{2}\sigma (1-\,\cos \,{{\theta }}_{a})$ ^49,50^. Interfacial surface energy causes the droplet to retract after spreading. The energy dissipation rate, ${\dot{E} }_{{\rm{diss}}}\propto \frac{\mu {v}^{2}{{\rm{R}}}_{{\rm{\max }}}^{2}}{h}$ shows that the viscous effects become more prominent with increasing impact velocity (*v*) (see Supplementary Table S1) and decreasing lamella thickness (*h*_lamella_). Therefore, viscosity causes the higher viscous dissipation of the droplet with high impact velocity, particularly at the recoiling phase, leading to a lower recoiling speed and increased recoiling time^41,51^ as observed in Fig. 2. This explains the eventual rise of the total contact times shown in Fig. 3. After the energy losses during spreading and retraction, if the droplets still have enough energy to depart, they bounce off and leave the surface.

### Effect of viscosity on dynamics of droplet impacting surfaces with macrotexture

**Figure 4:**
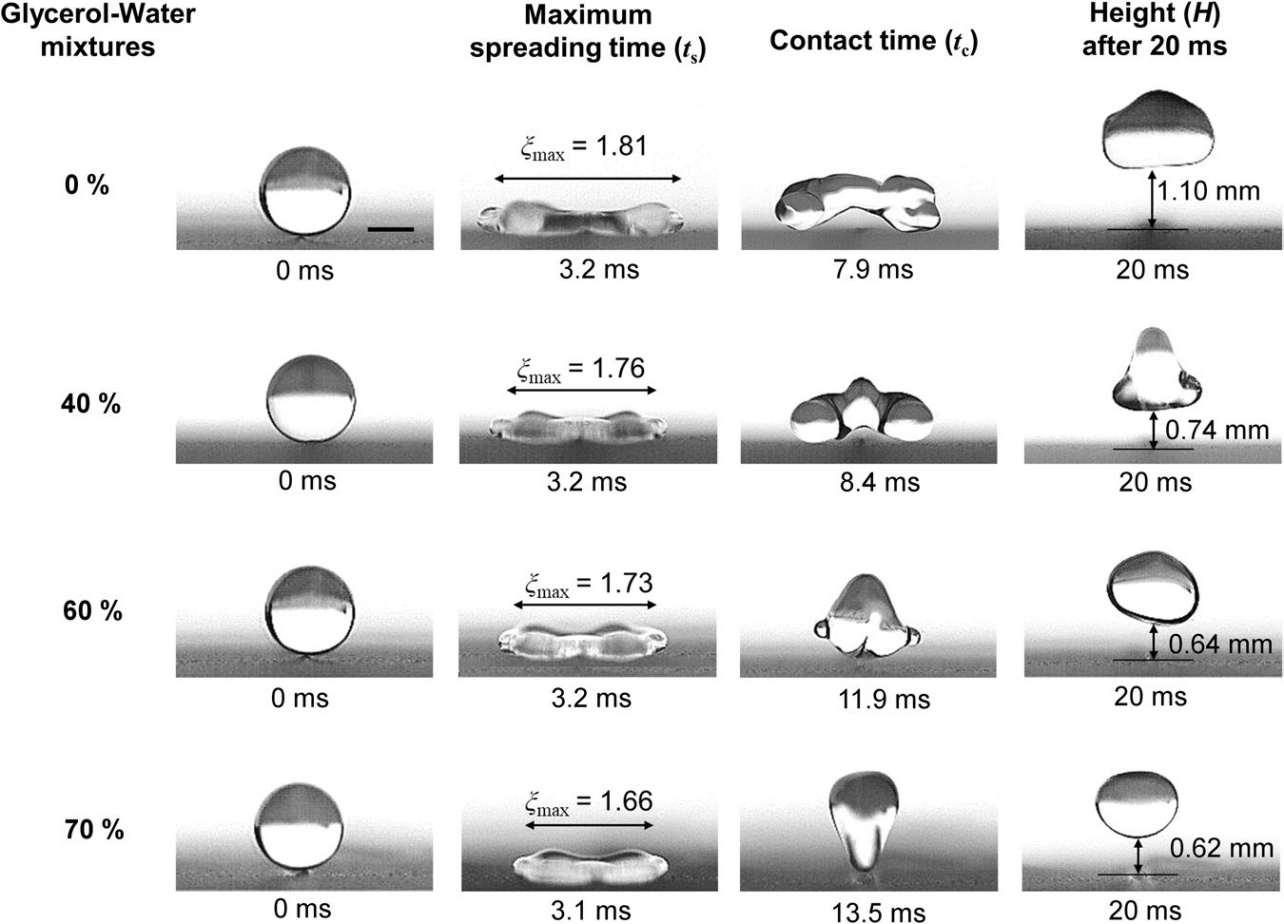
Effect of viscosity on dynamics of liquid droplet impacting macrotextured surface. Side views of various glycerol-water mixture (0%, 40%, 60%, and 70% by weight) droplets (radius, *R*_0_ = 1.3 mm) impacting and rebounding from the macrotextured superamphiphobic surface with impact velocity of 1 m/s (Weber number, *We* = 18~24). The spreading times are almost identical (~3.2 ms). For viscous liquids, the droplets spread less (2^nd^ column), bounce off with a higher contact times (3^rd^ column), and reach lower heights (*H*) at 20 ms after impact (4^th^ column) due to higher energy dissipation. For water, the droplets bounce off the surface faster along the macrotexture than the two side portions (left and right of the macrotexture) due to the buoyancy effect from the macrotexture. However, opposite incident occurs (side portions re-bounds earlier) for viscous liquids due to the higher viscous dissipation along the macrotexture. The scale bar is 1 mm.

**Figure 5:**
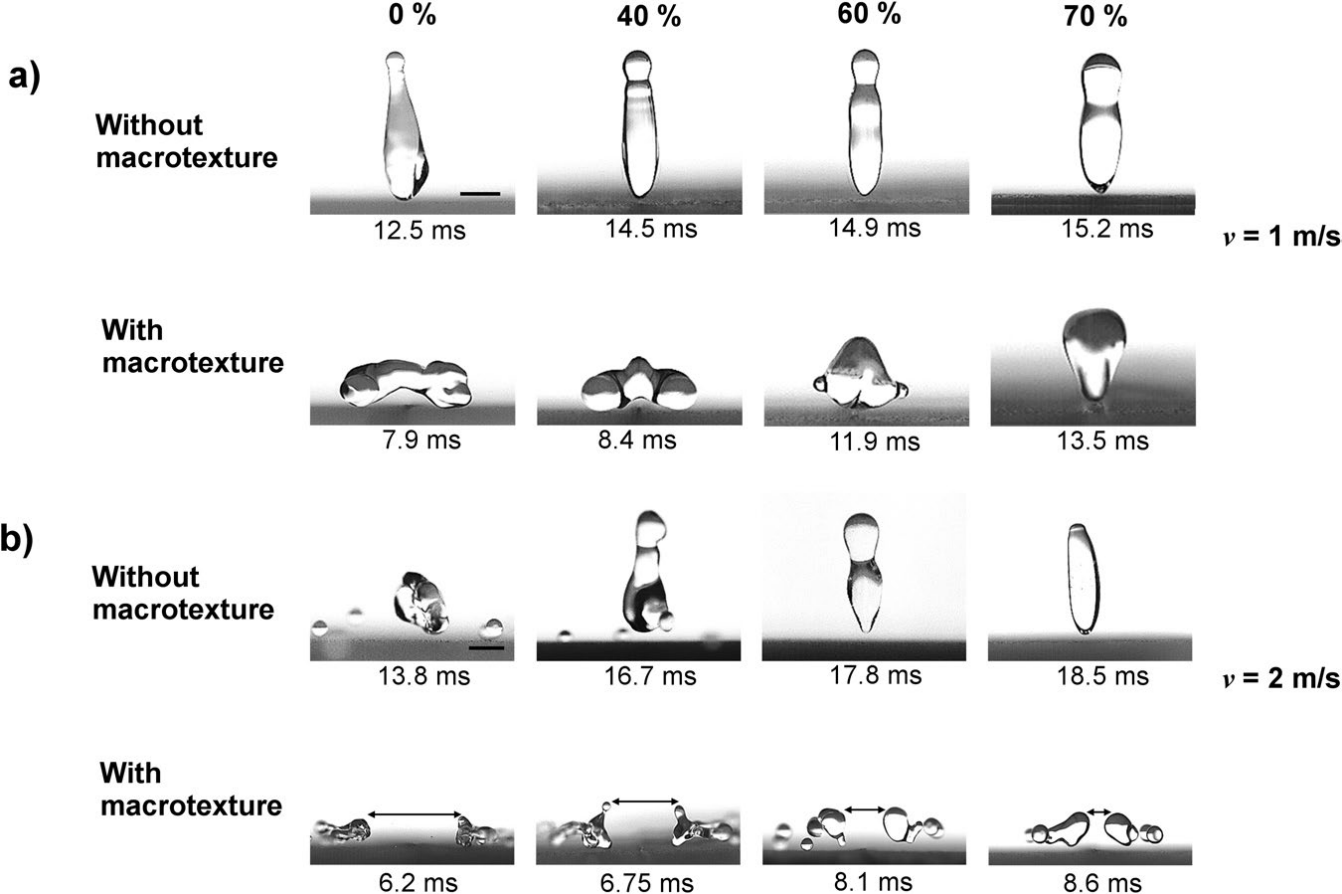
Modification of droplet dynamics and reduction of droplet contact time using a surface with a macrotexture. (**a**) Comparison of moments for droplets bouncing off the surfaces without (top) and with (bottom) macrotexture for various glycerol-water mixtures (0%, 40%, 60%, and 70% by weight) at impact velocity of 1 m/s (*We* = 18~24). Although the droplet contact time (*t*_c_) increases with higher viscosity for both surfaces (with and without macrotexture), the reduction in contact time (Δ*t*_c_) by macrotexture significantly decreases for viscous liquids at this low impact velocity. The scale bar is 1 mm. For full movies, see Supplementary Movie 1. (**b**) Comparison of moments for droplets bouncing off the surfaces without (top) and with (bottom) macrotexture for various glycerol-water mixtures at impact velocity of 2 m/s (*We* = 71~94). Droplets impacting the surface with a macrotexture at the high impact velocity (or Weber number) split after spreading and bounce off the surface faster than droplets impacting surfaces without the macrotexture. Contact time reduction (Δ*t*_c_) by macrotexture increases significantly for viscous liquid droplets at this high impact velocity, unlike what was observed at the low impact velocity. Moreover, on a surface with the macrotexture, the distance between the split droplets becomes closer as the droplet viscosity increases. Therefore, thin film retraction speed close to macrotexture decreases due to higher viscous dissipation effect induced by the macrotexture. The scale bar is 1 mm. For full movies, see Supplementary Movie 2.

To explore the dynamics of viscous droplets impacting macrotextures, we performed similar experiments on a superamphiphobic surface with the macrotexture at a wide range of impact velocities. The effect of viscosity on the dynamics of droplets impacting on macrotextured surfaces at a low impact velocity of *v* = 1 m/s (*We* = 18~24) is demonstrated in Fig. 4. The liquid droplet first impinges on the macrotexture rather than on the microtextured surface. For the various glycerol-water mixture droplets, the spreading times are almost identical (~3.2 ms), but the maximum spreading factor (*ξ*_max_ = *R*_max_/*R*_0_) decreases with increasing viscosity (2^nd^ column). The spreading is different than that observed when droplets impact surfaces without a macrotexture. The droplet experiences slightly less spreading along the macrotexture than across the macrotexture, whereas it experienced almost symmetrical spreading on the surfaces without macrotexture. The maximum spreading time on the macrotexture (~3.2 ms) is slightly higher than the spreading time of the same droplet impinging on the flat superamphiphobic surface (~3 ms) since the macrotexture deflects and expels the droplets, which feeds the lobes. The maximum spreading factor (*ξ*_max_) was found to be 1.81, 1.76, 1.73 and 1.66 for the 0%, 40%, 60% and 70% glycerol-water mixture droplets, respectively. The macrotexture modifies the dynamics of impacting droplet particularly after the spreading stage. At this low impact velocity, droplet splitting is not observed because of lower inertia and subsequently less spreading. Nevertheless, macrotexture induces buoyancy effect on droplet, pushing the center of the droplet upwards, eventually reducing the droplet contact time when compared to the contact time of the corresponding droplets impacting surfaces without a macrotexture. There are two divided subunits (not completely split) which are connected by a thin layer on the macrotexture as seen in Fig. 4 (second column), that explains the reduction of contact time without splitting. The water droplet bounces off the surface faster along the macrotexture than the two side portions (left and right of the macrotexture). However, the higher dissipation leads to the opposite incidents (side portions re-bound earlier) for viscous liquid droplets. The contact time (3^rd^ column) increases significantly and the height (*H*) 20 ms after impact decreases (4^th^ column) with higher viscosity. The droplet contact time were 7.9 ms, 8.4 ms, 11.9 ms and 13.5 ms for the increasing viscous droplets, respectively. The heights of the droplets (*H*) were found to be 1.1 mm, 0.74 mm, 0.64 mm, and 0.62 mm for 0%, 40%, 60%, and 70% glycerol-water mixture droplets, respectively. For the comparison, the moments for the droplet bouncing off the surfaces without (top) and with (bottom) macrotextures for various glycerol-water mixtures (0%, 40%, 60%, and 70% by weight) **(a)** at a low impact velocity of 1 m/s (*We* = 18~24) and **(b)** at a high impact velocity of 2 m/s (*We* = 71~94) are shown in Fig. 5. While studying a wide range of droplet impact velocities, 1 m/s is chosen as a low impact velocity at which significant viscous dissipation of liquid droplets are observed without splitting. Furthermore, 2 m/s is regarded as a high impact velocity at which significant changes in droplet contact time and dynamics along with viscous dissipation are observed, while avoiding splashing. At 1 m/s, the rise of droplet contact time due to increasing viscosity is more notable on the macrotextured surface compared to same on the surface without any macrotexture (Fig. 5a and see Supplementary Movie 1), which contradicts the purpose of using macrotextured surfaces. However, the droplet contact times on surfaces with the macrotexture are still less than the droplet contact times on surfaces without the macrotexture. This result which may at first seem counterintuitive can be clarified by understanding that contact area along the macrotexture is larger than the flat surface. The higher contact area causes higher energy dissipation along the macrotexture. However, the viscous dissipations are less significant at the lower impact velocity of 1 m/s for droplets impacting on flat surfaces. Therefore, the contact time reduction (Δ*t*_c_, which is the difference between the contact times of the similar droplets impacting surfaces with and without macrotexture) decreases significantly at this low impact velocity when viscosity increases as shown in Fig. 5a.

On the other hand, the viscous dissipation is more prominent at the higher impact velocity of 2 m/s as mentioned earlier, so viscous liquid droplets show significant increase in contact time when bouncing off the flat superamphiphobic surfaces (Fig. 5b top). However, the recoiling stage of a droplet impacting on the macrotexture at high impact velocity is quite different (Fig. 5b bottom). The macrotexture causes the droplet to split after spreading, pushes the split drops away, leading the dewetting dynamics of the droplet to be quite different than what was observed on surfaces without the macrotextures. Moreover, at this higher impact velocity *v* = 2 m/s (*We* = 71~94), splashing is observed for 0% and 40%, but not for 60% and 70% glycerol-water mixtures, due to suppression of the splashing by viscosity^52,53^ (Fig. 5b, top). The droplet contact times on the flat surface are 13.8 ms, 16.7 ms, 17.8 ms, and 18.5 ms for 0%, 40%, 60%, and 70% glycerol-water mixture droplets, respectively. However, the droplet impinging on the macrotexture (Fig. 5b, bottom) bounces off the surface faster than the droplet impinging on the surface without any macrotexture. The contact time of the droplets on the superamphiphobic macrotexture are 6.2 ms, 6.75 ms, 8.1 ms, and 8.6 ms for 0%, 40%, 60%, and 70% glycerol-water mixture droplets, respectively (see Supplementary Movie 2). Therefore, the contact time reduction (Δ*t*_c_) by macrotexture increases significantly for viscous liquid droplets at this high impact velocity, unlike what was observed with the similar droplets at low impact velocity. Thus, viscous effect on bouncing droplet is superseded by the macrotexture at high impact velocities in respect of the contact times. Moreover, the distance between two split droplets at the moment of leaving the surface are shown in Fig. 5b (bottom), which decreases with the droplet viscosity. For a viscous droplet, the thin film retraction speed close to the macrotexture decreases due to the higher viscous dissipation along the macrotexture as mentioned earlier. In addition, this also substantiates the argument for the increasing values of contact time of viscous liquid droplets impacting surfaces with the macrotexture at a low impact velocity.

**Figure 6:**
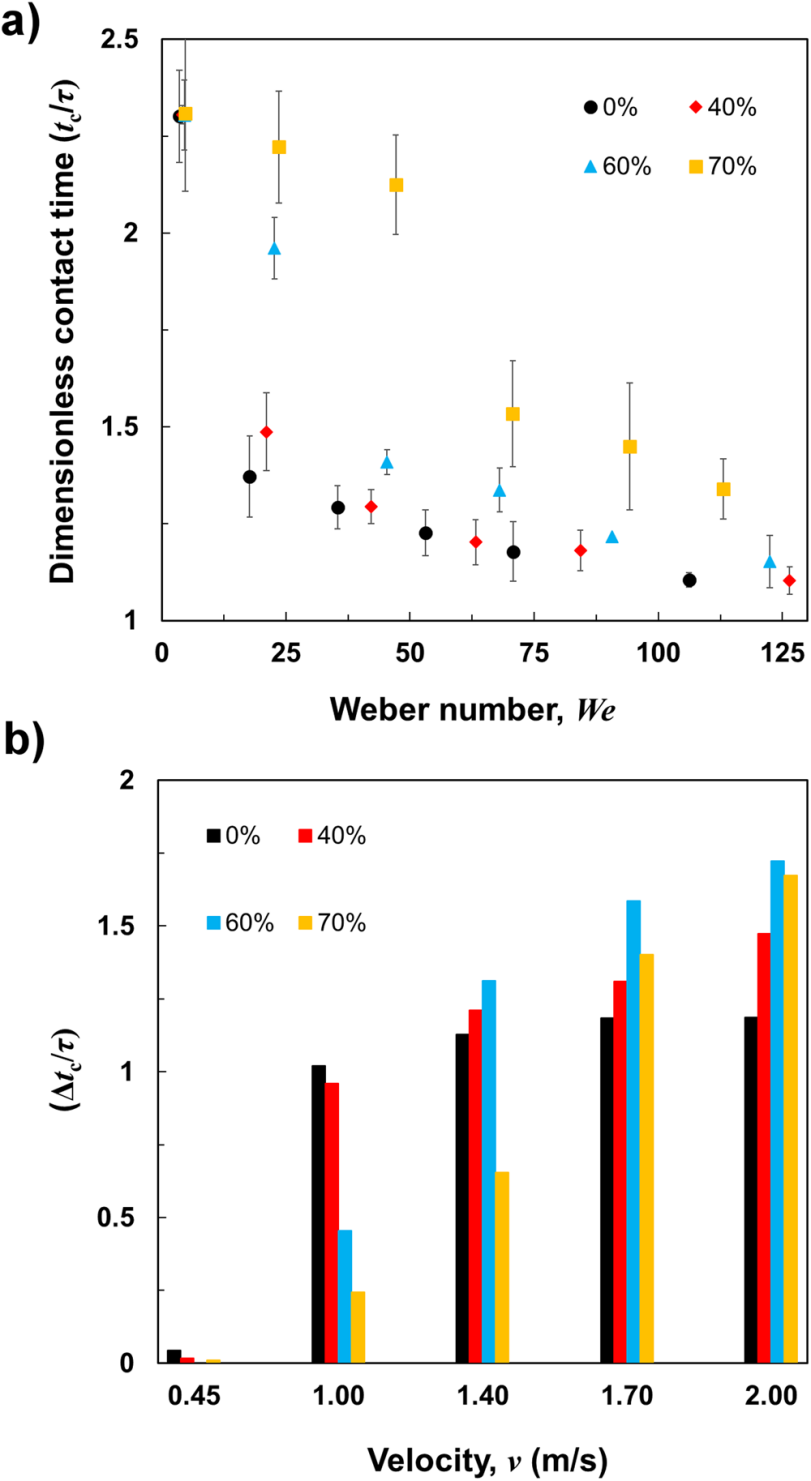
Reduction in contact time by macrotexture. (**a**) Dimensionless contact time (*t*_c_/*τ*) of various glycerol-water mixture droplets (*R*_0_ = 1.3 mm) impacting on the macrotexture, as a function of Weber number (*We*). Error bars represent the standard deviation of the experimental values. (**b**) Dimensionless contact time reduction (Δ*t*_c_/*τ*) due to the macrotexture for various glycerol-water mixture droplets as a function of impact velocity *v*, where Δ*t*_c_ is the difference between the contact times of the similar droplets impacting the surface with and without the macrotexture.

We further investigated dynamics of various viscous droplets impacting surfaces with the macrotexture for a wide range of impact velocities. The dimensionless contact times (*t*_c_/*τ*) of various glycerol-water mixture droplets bouncing off the macrotexture with different Weber numbers (*We*) are shown in Fig. 6a. For the very low impact velocity of 0.45 m/s (*We* = 3 to 5), almost identical dimensionless contact time (*t*_c_/*τ* = 2.3) is observed for droplets of the different viscosities. At this low impact velocity, the droplet is neither split nor has significant upward buoyancy from the macrotexture, and ultimately exhibits almost similar bouncing dynamics to the droplet bouncing on a surface without a macrotexture. We showed earlier (Fig. 3b) that the contact time of liquid droplets with various viscosities bouncing off superamphiphobic surface without the macrotexture increases with higher Weber numbers. In contrast, the contact time of liquid droplets with various viscosities bouncing off the macrotexture decreases with higher Weber numbers (Fig. 6a). To study the difference between the contact times of a droplet with similar impact velocity impinging on a superamphiphobic surface with and without macrotexture, we plot the dimensionless contact time reduction (Δ*t*_c_/*τ*) by macrotexture for various glycerol-water mixtures (0%, 40%, 60%, and 70% by weight) as a function of velocity (Fig. 6b). At the low impact velocity (*v* = 0.45 m/s), no significant reduction in contact time by the macrotexture was observed for droplets with any viscosity. As the impact velocity increases, the reduction in contact time by the macrotexture increases for all viscous liquid droplets. Due to the opposing trend of contact times for viscous liquid droplets on surfaces with and without macrotextures, the reduction in contact time keeps increasing with higher impact velocity (or Weber number). Although the reduction in contact time is increasing with impact velocity, there are certain threshold velocities for different viscosities for which sudden jumps in the contact time reduction are found. There are sudden jumps in contact time reductions from impact velocity 0.45 m/s to 1.0 m/s for both 0% and 40% glycerol-water mixtures (Fig. 6b). For 60% and 70% glycerol-water mixtures, this significant reduction occurs from impact velocity 1.0 m/s to 1.40 m/s and from 1.40 m/s to 1.70 m/s, respectively. After these threshold velocities, the contact time reduction values increase in a steady manner. While investigating the threshold velocity for this significant jump in contact time reduction, we found that the droplet experiences a transition from non-splitting to splitting between those two impact velocities. When the droplet has kinetic energy such that it is very close to split but does not exhibit complete splitting eventually, the droplet forms four lobes after spreading.

## Discussion

In a broad perspective, the macrotexture helps the incoming droplets bounce off the surface faster than the surface without macrotexture. However, if the droplet after spreading becomes substantially thicker than the macrotexture size, there is no considerable effect by the macrotexture on the droplet dynamics. This occurs when the macrotexture size is very small and/or the impact velocity of the droplet is very low. With a macrotexture size of 50 µm in diameter, no reduction of contact time for a droplet with radius of 1.4 mm was observed in agreement with Gauthier *et al*.^40^. Considering all these facts, the macrotexture with a diameter of *a* = 150 µm was chosen which was appropriate for achieving the reduced contact times for incoming droplets with radius *R*_0_ = 1.3 mm in a wide regimes of impact velocities and viscosities. Moreover, it was difficult to observe bouncing phenomena for viscous droplets with the same droplet size by a larger macrotexture (a ~ 400 µm) in our preliminary experiments.

The percentage of contact time reduction (Δ*t*_c_/*t*_0_) by macrotexture decreased from ~35% to ~10% due to the changes in droplet viscosity from 1 mPa.s to 22.5 mPa.s at low impact velocity of *v* = 1 m/s (Fig. 5a). At the high impact velocity of *v* = 2 m/s (Fig. 5b), macrotexture could compensate the viscous effect, making the percentage of contact time reduction (Δ*t*_c_/*t*_0_) remain almost same (~55%) for the droplets with higher viscosities. At low impact velocity, water droplets do no split but bounce off quickly due to the buoyancy effect induced by the macrotexture. When the viscosity of the droplet is higher, there is significant loss of energy on the macrotexture-droplet contact area, leading to a delay in rebounding of the droplet from the macrotexture. Therefore, for the non-splitting droplet with low inertial force, macrotexture gradually induces higher contact times for higher viscous liquids. When the impact velocity is high enough, the droplets split during and/or after spreading. For each of the split droplets, de-wetting starts from both the macrotexture and the droplet edge. As the recoiling distance after splitting is half of the recoiling distance without splitting^22^ and also the four lobes follow to form two split droplets^40^, macrotexture reduces the contact time almost to half. Even if there is viscous dissipation due to the higher droplet contact area on the macrotexture, the split droplets de-wet the macrotexture at very first stage of the recoiling. These generate less of a contact time increase of the droplet on superamphiphobic surface with macrotexture when compared to the significant contact time increase on the flat superamphiphobic surface.

As the previous explanations regarding contact time reduction of water droplet do not hold for the viscous liquid droplets anymore, we searched for a new condition at which the macrotexture affects the contact time significantly. If the spreading film is thicker than the size of the macrotexture, then the droplet does not split, leading to limited even nullified effect on the contact time^40^. To investigate the minimum impact velocity (*v*_MS_) at which the droplet can split by the macrotexture, we treat the droplet as a circular cylindrical disc with thickness of *h* at maximum spreading. Consequently, this thickness *h* depends on the maximum spreading of the droplet (*R*_max_). Considering the droplet with spherical shape (radius *R*_0_) before impacting and cylindrical shape at maximum spreading (radius *R*_max_), conservation of mass theory yields the relation:

6 $${\xi }_{{\rm{\max }}}^{2}=\frac{{R}_{{\rm{\max }}}^{2}}{{R}_{0}^{2}}=\frac{4{R}_{0}}{3h}$$

Recently, Zhao *et al*. have proposed a model of maximum spreading factor for various viscous liquid droplets on superamphiphobic surface^54^. Based on energy conservation, they developed the following relation:

7 $${\xi }_{{\rm{\max }}}=\frac{2}{3}\sqrt{\frac{2(We+6)}{\frac{We}{\sqrt{2Re}}+1-\,\cos \,{\theta }_{{\rm{eq}}}}}\,\cos \,\alpha |\alpha =\frac{1}{3}\arccos (18\sqrt{\frac{\frac{We}{\sqrt{2Re}}+1-\,\cos \,{\theta }_{eq}}{2{(We+6)}^{3}}})$$

where *Re* is the Reynolds number (*Re* = *ρvR*_0_/*µ*) and cos*θ*_eq_ is the equilibrium contact angle of the corresponding liquid droplet on the superamphiphobic surface.

It was initially agreed that the thickness (*h*) of the droplet at maximum spreading moment should be less than the macrotexture size (*a*) to split the droplet. However, the droplet shows splitting even when the thickness at the maximum spreading moment is greater than the macrotexture size. From the experiments, it was observed that the thickness of the center of the droplet continues to decrease, still feeding the rim of the droplet for a short time after it reaches to the maximum spreading. Thus, when the macrotexture can cut off at least 70% thickness of the droplet during maximum spreading stage, it continues to separate until the droplet completely split and eventually forms two smaller split droplets during the beginning of recoiling phase. Considering that, the new criteria for having the droplet split follows the empirical relation:

8 $$h\le {a}_{{\rm{effective}}}|{a}_{{\rm{effective}}}\approx \frac{7}{5}a$$

Combining the above relations (see Supplementary Information: Reasoning for Model), we developed a new model for predicting the minimum impact velocity (*v*_MS_) of the droplet for splitting:

9 $$\frac{\rho {R}_{0}\,{\cos }^{2}\alpha }{\sigma }{v}^{2}-\frac{15{R}_{0}\sqrt{\rho {R}_{0}\mu }}{14\sqrt{2}a\sigma }{v}^{\frac{3}{2}}-\frac{15{R}_{0}}{14a}(1-\,\cos \,{\theta }_{eq})+6\,{\cos }^{2}\alpha =0$$

**Figure 7:**
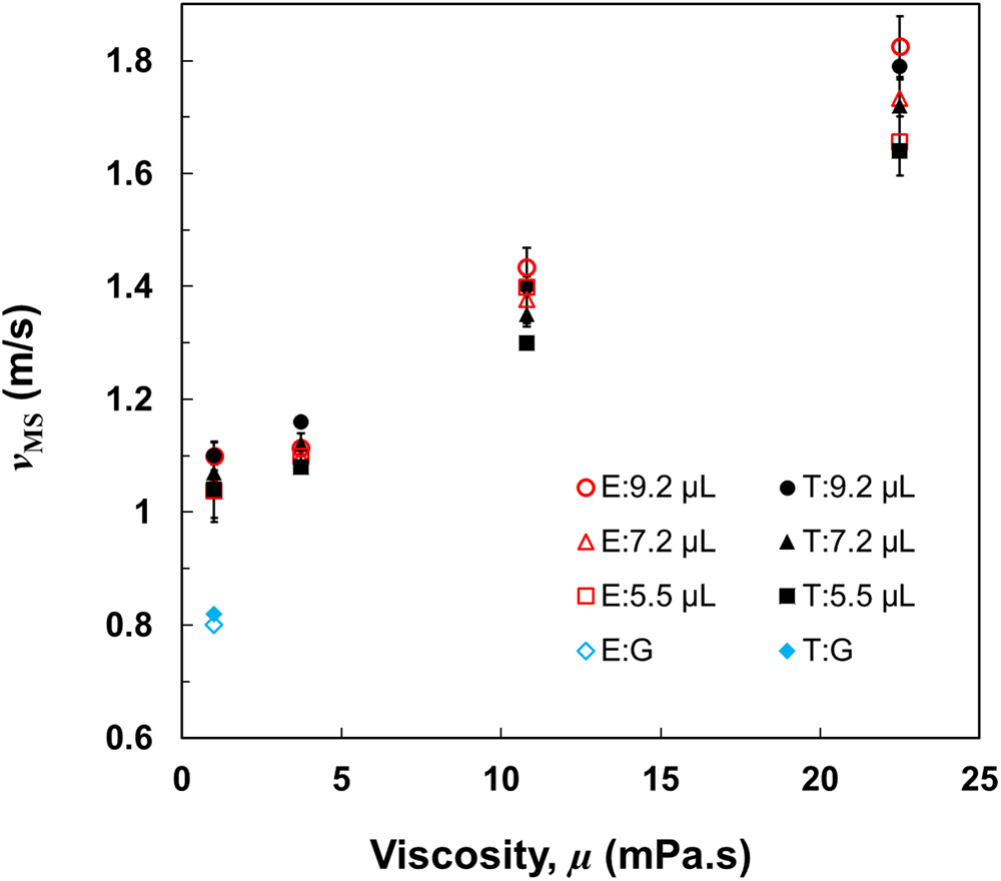
Minimum impact velocity for splitting. Comparison between experimental (red hollow) and theoretical (black solid) values of minimum impact velocity to split (*v*_MS_) for droplets with various viscosities and volumes (9.2, 7.2, 5.5 µL). The blue boxes represent the values collected and assumed from the works of Gauthier *et al*.^40^. The viscous droplets spread less due to the higher energy dissipation during spreading, leading to the higher minimum impact velocity needed for splitting. The theoretical data from the developed model agrees well with the experimental data. Error bars represent the standard deviation of the experimental values.

For the validation of our model, we performed experiments to measure the minimum impact velocity (*v*_MS_) for splitting of liquid droplets with different viscosities. The plots in Fig. 7 compare experimental (red hollow) and theoretical (black solid) values of minimum impact velocity for splitting (*v*_MS_) with different droplet volumes (9.2, 7.2, 5.5 µL) on macrotexture with size of *a* = 150 µm. All the corresponding values of impacting velocity for splitting are mentioned in Table 2 as well. The experimental values of *v*_MS_ for the various liquid droplets impacting on a different size of the macrotexture (*a* = 180 µm) are also measured and compared with the values obtained from our model (Supplementary Table S2). The minimum impact velocity for splitting (*v*_MS_) increases with the viscosity of the droplet (Fig. 7) as the viscous droplets spread less and consequently have larger thickness at maximum spreading moment. Therefore, a viscous droplet needs a higher impact velocity for achieving the desired thickness to split as verified by both the experimental and predicted theoretical values. To compare with previously reported result, the collected data (*R*_0_ = 1.3 mm, *a* = 200 µm, *θ*_eq_ ~ 160°) from the work of Gauthier *et al*.^40^ are used as input in our proposed model. The minimum impact velocity for splitting (*v*_MS_) is not reported by Gauthier *et al*., but it is fair to assume the value to be slightly higher than the impact velocity at which the maximum reduction of droplet contact time started. In our case, the difference is about 0.1 m/s for the water droplet. Therefore, the impact velocity for splitting is assumed to be ~0.8 m/s which is close to the predicted value (0.84 m/s) obtained from the model. Comparisons between experimental and theoretical values verify that the predicting model not only provides the correct scaling behavior for the minimum impact velocity at which the droplet splits, but also provides a very close estimation of the numerical values. The slight differences might be due to the assumption of the droplet being in complete cylindrical shape, the imperfection of the empirical relation, and some minor potential experimental errors.

**Table 2:** Comparison between theoretical and experimental values of minimum impact velocities of various glycerol-water mixture (0%, 40%, 60%, and 70% by weight) droplets to split with different volumes (9.2, 7.2, and 5.5 µL). Macrotexture size, *a* = 150 µm.

Glycerol weight (%)	Droplet volume (µL)	Minimum impact velocity for splitting, *v*_MS_ (m/s)	
		Theoretical	Experimental
0	9.2	1.09	1.07 ± 0.03
	7.2	1.07	1.05 ± 0.03
	5.5	1.04	1.05 ± 0.04
40	9.2	1.15	1.14 ± 0.07
	7.2	1.12	1.10 ± 0.03
	5.5	1.08	1.10 ± 0.03
60	9.2	1.38	1.43 ± 0.03
	7.2	1.33	1.40 ± 0.05
	5.5	1.27	1.32 ± 0.03
70	9.2	1.78	1.82 ± 0.05
	7.2	1.70	1.74 ± 0.04
	5.5	1.60	1.64 ± 0.03

**Figure 8:**
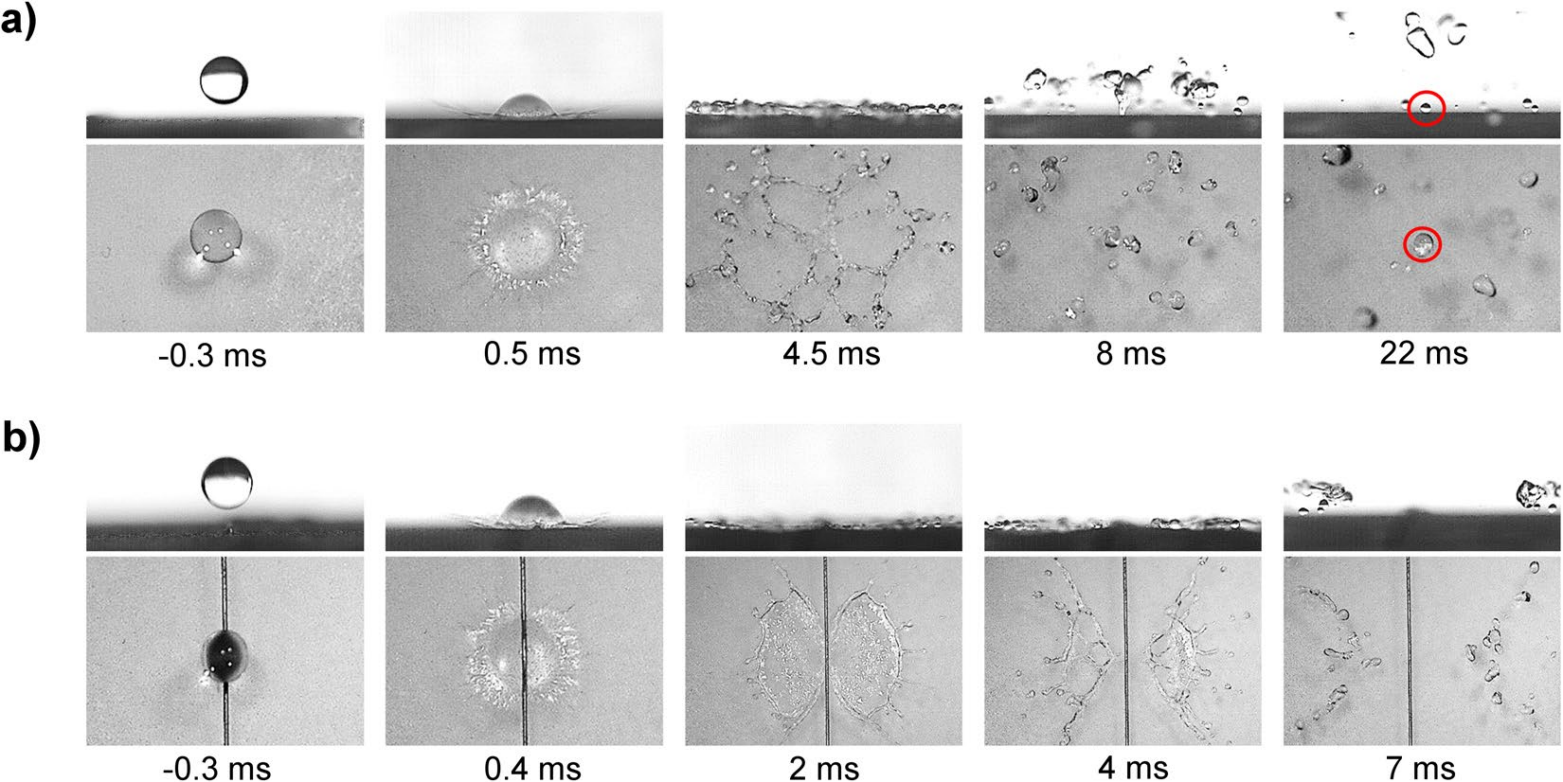
Avoiding droplet impalement at critical impact velocity by macrotexture. Side and top views of water droplet (*R*_0_ = 1.3 mm) with very high impact velocity, *v* = 3.37 m/s (*We* ~ 206) impinging on superamphiphobic surface without and with the macrotexture. (**a**) A tiny part of the droplet sticks to the surface after bouncing which shows the impalement of the water droplet to the surface without macrotexture at the critical impact velocity (*v*_c_). (**b**) On a surface with the macrotexture the similar droplet avoids the impalement, being cut and moving in two different directions due to their interfacial energies and expulsion from the macrotexture. For full movies, see Supplementary Movie 3.

Furthermore, we investigate the critical impact velocity (*v*_c_) on the superamphiphobic surface with and without macrotexture. For water, the critical impact velocity is found to be *v* = 3.37 m/s (*We* = 113) on the surface. The impalement of the water droplet occurs where the wetting pressures overcome the capillary pressure and the Wenzel drop is unable to rebound fully from the surface (Fig. 8a). We also noticed that the size of the Wenzel drops increases with the impacting velocity above critical impact velocity (*v*_c_). On the flat superamphiphobic surface, the center of the droplet is the last part to rebound. However, on the macrotexture, a similar droplet with the same impact velocity splits and the center of the droplet starts recoiling and rebounding just after maximum spreading (Fig. 8b). The split drops de-wet the macrotexture at the very beginning of the recoiling stage, when the interfacial surface energy is still very high. Therefore, the split drops can eventually rebound without any tiny droplet left behind at the center by sticking as observed with a similar droplet on surface without the macrotexture. The impalement avoiding incidents by the macrotexture at critical impact velocity for 0%, 40%, 60%, and 70% glycerol-water mixture droplets are shown in Supplementary Movie 3. Macrotexture avoids the impalements for a certain range of impact velocity above the critical impact velocity (*v*_c,_ which is defined for flat superamphiphobic surface) for liquids with the various viscosities i.e. macrotexture can increase the critical impact velocity of a surface.

In conclusion, we demonstrated the role of viscosity for droplets impacting on superamphiphobic surface with the macrotexture for a wide range of impact velocity. Total contact time of a liquid droplet impacting at a low velocity is reduced by the uplifting buoyancy from the macrotexture. However, the contact time reduction (Δ*t*_c_) by the macrotexture decreases significantly if the viscosity of the liquid droplet increases. When the droplet does not split at a low impact velocity, the macrotexture may increase the contact time of a viscous droplet, which contradicts with the purpose of using macrotextured surfaces for applications that involved viscous liquids, such as preventing ice formation during freezing rain or enabling high resolution during inkjet printing. On the contrary, when the impact velocity is high enough to split the droplet, the reduction in contact time significantly increases with an in increase in droplet viscosity. Therefore, macrotexture can compensate the viscous dissipation of a liquid droplet impacting on superamphiphobic surfaces with high impact velocities. This work introduced the idea of the threshold impact velocity for non-splitting to splitting transition of the droplets which eventually determines the significant reductions in contact times. Considering the importance of the transition from non-splitting to splitting droplet, a novel model for predicting the minimum impact velocity, above which the droplet is expected to split, is developed. Moreover, we demonstrated that the critical impact velocity of a viscous liquid droplet impacting superamphiphobic surface can be raised by using the macrotexture. These findings are of potential importance to enable engineering surfaces for achieving maximized repellency of the freezing rain and viscous liquids such as oils for various industrial applications.

## Methods

### Surface preparation and characterization

Silicon substrates were rinsed with fresh water and allowed to thoroughly dry. A tinned-copper wire of diameter, *a* = 150 µm was placed on flat silicon substrates. The samples were then treated with a commercial spray (Rust-Oleum® NeverWet® Liquid Repelling Treatment), which deposited an anti-wetting solution. The sprayed samples were placed under a fume hood at lab temperature (~22 °C) for 5 hours to dry. After the solvent of the spray (acetone) evaporated, microparticles coated both the substrate and the wire, resulting in a macrotextured liquid-repelling surface. A FEI QUANTA 3D FEG SEM was used for obtaining the images of the coated silicon substrates. The samples were coated with 10 nm of gold (Ted Pella 108 Manual Sputter Coater) and then images were obtained using a 2-kV acceleration voltage.

### Viscous liquid preparation

To make various glycerol-water mixtures (0%, 40%, 60%, and 70% by weight), deionized (DI) water (SIGMA-ALDRICH) and glycerol (GX0190 - EMD Millipore) were mixed together and made into solutions. A magnetic stirrer (BIPEE SH-2) was used to make the homogeneous solutions. A digital analytical balance scale (U.S. Solid, USS-DBS8) was used to measure the weights of the liquids accurately (repeatability of ±0.1 mg).

### Droplet impact experiment

The experimental setup is shown in Supplementary Fig. 1. The superamphiphobic surface was placed on a horizontal stage, above which 9.2 μL droplets (radius, *R*_0_ = 1.3 mm) were generated by pumping liquids through a steel needle using a ramé-hart Automated Dispensing System (p/n 100–22) with accuracy of ±0.002 µL. Throughout the experiments, two needles (26 and 24 gauges with inside diameter (ID) of 0.256 mm and 0.305 mm) were used for water and glycerol-water mixture (40%, 60%, and 70%) droplet release, respectively. The droplet falls on the macrotexture (size, *a* = 150 µm) with impact velocity *v*. Drop Volume Control software was used to control the liquid input and output. A high-speed video camera (OLYMPUS i-SPEED TR) was used to capture slow motion impacts of the droplets at 10,000 frames per second (FPS). Both Fiji-ImageJ and i-SPEED Viewer (iX CAMERAS) were used for analyzing the droplet dynamics and measuring the maximum spreading (*R*_max_) and thickness (*h*). Impact velocity, *v* is varied from 0.4 m/s to 3.8 m/s by adjusting the droplet releasing heights and verified via the video analyzing software. For repeatability, each impact experiment was repeated at least five times and droplet contact times (*t*_c_) were averaged from multiple videos. For measuring the contact time of split droplets on the macrotexture, contact times of the two subunit droplets were recorded and averaged.

### Contact angle measurement

The static (*θ*_s_), advancing (*θ*_a_), and receding (*θ*_r_) contact angles of the liquid droplets were measured on the superamphiphobic surfaces using the sessile drop technique with the help of contact angle tool from Fiji–ImageJ software. 5 µL droplets was deposited using the ramé-hart Automated Dispensing System (p/n 100–22) for static contact angle measurements. The advancing and receding contact angles were measured by depositing a water droplet of 10 µL on the surface, then increasing the volume by 2 µL increments until advancement in the liquid meniscus was observed and then decreasing the volume by the same rate until receding motion was seen. Advancing contact angles were considered as the maximum angles observed during the droplet growth, while receding contact angles were calculated from the drop profile just before the interface receded. To ensure repeatability, each contact angle value was averaged from measurements of ten droplets distributed across the sample. These measurements are performed at general laboratory environmental conditions (temperature of ~22 °C and relative humidity of ~40%).

## Electronic supplementary material

Supplementary Information

Supplementary Movie 1

Supplementary Movie 2

Supplementary Movie 3

## References

[1] Massinon M, Lebeau F (2012). Experimental method for the assessment of agricultural spray retention based on high-speed imaging of drop impact on a synthetic superhydrophobic surface. Biosystems Engineering.

[2] Smith D, Askew S, Morris W, Shaw D, Boyette M (2000). Droplet size and leaf morphology effects on pesticide spray deposition. Transactions of the ASAE.

[3] Rioboo Romain, Tropea Cameron, Marengo Marco (2001). OUTCOMES FROM A DROP IMPACT ON SOLID SURFACES. Atomization and Sprays.

[4] van der Bos A (2014). Velocity profile inside piezoacoustic inkjet droplets in flight: comparison between experiment and numerical simulation. Physical review applied.

[5] Yarin AL (2006). Drop impact dynamics: splashing, spreading, receding, bouncing…. Annu. Rev. Fluid Mech..

[6] Mishchenko L (2010). Design of Ice-free Nanostructured Surfaces Based on Repulsion of Impacting Water Droplets. Acs Nano.

[7] Bergeron V, Bonn D, Martin JY, Vovelle L (2000). Controlling droplet deposition with polymer additives. Nature.

[8] Yao H, Zhou X (2018). A Pioneering Method for Reducing Water Droplet Erosion. Journal of Fluids Engineering.

[9] Maitra T (2014). On the Nanoengineering of Superhydrophobic and Impalement Resistant Surface Textures below the Freezing Temperature. Nano Letters.

[10] Meuler AJ, McKinley GH, Cohen RE (2010). Exploiting topographical texture to impart icephobicity. ACS nano.

[11] Blossey R (2003). Self-cleaning surfaces—virtual realities. Nature materials.

[12] Lu Y (2015). Robust self-cleaning surfaces that function when exposed to either air or oil. Science.

[13] Wisdom KM (2013). Self-cleaning of superhydrophobic surfaces by self-propelled jumping condensate. Proceedings of the National Academy of Sciences.

[14] Quéré D (2005). Non-sticking drops. Reports on Progress in Physics.

[15] Sojoudi H, Wang M, Boscher N, McKinley G, Gleason K (2016). Durable and scalable icephobic surfaces: similarities and distinctions from superhydrophobic surfaces. Soft Matter.

[16] Sojoudi H, McKinley GH, Gleason KK (2015). Linker-free grafting of fluorinated polymeric cross-linked network bilayers for durable reduction of ice adhesion. Materials Horizons.

[17] Sojoudi H (2018). Scalable and durable polymeric icephobic and hydrate-phobic coatings. Soft Matter.

[18] Sojoudi H, Walsh MR, Gleason KK, McKinley GH (2016). Designing Durable Vapor-Deposited Surfaces for Reduced Hydrate Adhesion. Advanced Materials Interfaces.

[19] Nakajima A, Hashimoto K, Watanabe T (2001). Recent studies on super-hydrophobic films. Monatshefte für Chemie/Chemical Monthly.

[20] Jung YC, Bhushan B (2008). Dynamic effects of bouncing water droplets on superhydrophobic surfaces. Langmuir.

[21] Richard D, Clanet C, Quéré D (2002). Surface phenomena: Contact time of a bouncing drop. Nature.

[22] Bird JC, Dhiman R, Kwon H-M, Varanasi KK (2013). Reducing the contact time of a bouncing drop. Nature.

[23] Liu Y (2014). Pancake bouncing on superhydrophobic surfaces. Nature Physics.

[24] Schutzius TM (2015). Spontaneous droplet trampolining on rigid superhydrophobic surfaces. Nature.

[25] Quan Y, Zhang L-Z (2014). Numerical and analytical study of the impinging and bouncing phenomena of droplets on superhydrophobic surfaces with microtextured structures. Langmuir.

[26] Shen Y (2015). Relationship between wetting hysteresis and contact time of a bouncing droplet on hydrophobic surfaces. ACS applied materials & interfaces.

[27] Moqaddam AM, Chikatamarla SS, Karlin IV (2017). Drops bouncing off macro-textured superhydrophobic surfaces. Journal of Fluid Mechanics.

[28] Liu Y, Whyman G, Bormashenko E, Hao C, Wang Z (2015). Controlling drop bouncing using surfaces with gradient features. Applied Physics Letters.

[29] Chen L, Wu J, Li Z, Yao S (2011). Evolution of entrapped air under bouncing droplets on viscoelastic surfaces. Colloids and Surfaces A: Physicochemical and Engineering Aspects.

[30] Bouwhuis W (2012). Maximal air bubble entrainment at liquid-drop impact. Physical review letters.

[31] Sojoudi H (2017). Stable Wettability Control of Nanoporous Microstructures by iCVD Coating of Carbon Nanotubes. ACS Applied Materials & Interfaces.

[32] Liu K, Tian Y, Jiang L (2013). Bio-inspired superoleophobic and smart materials: design, fabrication, and application. Progress in Materials Science.

[33] Chu Z, Seeger S (2014). Superamphiphobic surfaces. Chemical Society Reviews.

[34] Bartolo D (2006). Bouncing or sticky droplets: Impalement transitions on superhydrophobic micropatterned surfaces. EPL (Europhysics Letters).

[35] Chandra S, Avedisian C (1991). On the collision of a droplet with a solid surface. Proc. R. Soc. Lond. A.

[36] Deng T (2009). Nonwetting of impinging droplets on textured surfaces. Applied Physics Letters.

[37] Schutzius TM (2014). Physics of icing and rational design of surfaces with extraordinary icephobicity. Langmuir.

[38] Bahadur V, Garimella SV (2009). Preventing the Cassie− Wenzel transition using surfaces with noncommunicating roughness elements. Langmuir.

[39] Bird RB (2002). Transport phenomena. Applied Mechanics Reviews.

[40] Gauthier, A., Symon, S., Clanet, C. & Quéré, D. Water impacting on superhydrophobic macrotextures. *Nature communications* **6** (2015). 10.1038/ncomms9001 PMC4918367 26259509

[41] Maitra T (2014). Supercooled water drops impacting superhydrophobic textures. Langmuir.

[42] Reyssat M, Richard D, Clanet C, Quéré D (2010). Dynamical superhydrophobicity. Faraday discussions.

[43] Patterson CJ, Shiri S, Bird JC (2016). Macrotextured spoked surfaces reduce the residence time of a bouncing Leidenfrost drop. Journal of Physics: Condensed Matter.

[44] Abolghasemibizaki M, Mohammadi R (2018). Droplet impact on superhydrophobic surfaces fully decorated with cylindrical macrotextures. Journal of colloid and interface science.

[45] Deng X, Schellenberger F, Papadopoulos P, Vollmer D, Butt H-JR (2013). Liquid drops impacting superamphiphobic coatings. Langmuir.

[46] Clanet C, Béguin C, Richard D, Quéré D (2004). Maximal deformation of an impacting drop. Journal of Fluid Mechanics.

[47] Chen L, Xiao Z, Chan PC, Lee Y-K, Li Z (2011). A comparative study of droplet impact dynamics on a dual-scaled superhydrophobic surface and lotus leaf. Applied surface science.

[48] Mao T, Kuhn D, Tran H (1997). Spread and rebound of liquid droplets upon impact on flat surfaces. AIChE Journal.

[49] Pasandideh‐Fard M, Qiao Y, Chandra S, Mostaghimi J (1996). Capillary effects during droplet impact on a solid surface. Physics of fluids.

[50] Carey, V. P. Liquid-vapor phase-change phenomena. *Taylor and Francis*, Bristol, PA (1992).

[51] Duvivier D, Seveno D, Rioboo R, Blake T, De Coninck J (2011). Experimental evidence of the role of viscosity in the molecular kinetic theory of dynamic wetting. Langmuir.

[52] Stevens CS, Latka A, Nagel SR (2014). Comparison of splashing in high-and low-viscosity liquids. Physical Review E.

[53] Schaarsberg MHK (2016). From splashing to bouncing: The influence of viscosity on the impact of suspension droplets on a solid surface. Physical Review E.

[54] Zhao B, Wang X, Zhang K, Chen L, Deng X (2016). Impact of Viscous Droplets on Superamphiphobic Surfaces. Langmuir.

[55] Association, G. P. *Physical properties of glycerine and its solutions*. (Glycerine Producers’ Association, 1963).

[56] Romero CM, Paéz MS (2006). Surface tension of aqueous solutions of alcohol and polyols at 298.15 K. Physics and Chemistry of Liquids.

