# Supplementary material for: Effect of superamphiphobic macrotextures on dynamics of viscous liquid droplets
Source: Sci Rep. 2018 Oct 18;8:15344. doi: 10.1038/s41598-018-33656-9 (PMC6194085; doi:10.1038/s41598-018-33656-9)
Supplement: Supplementary file 1 — Supplementary Information [file 41598_2018_33656_MOESM1_ESM.pdf]

Supplementary Information

Effect of superamphiphobic macrotextures on dynamics of viscous liquid droplets

Asif Raiyan[1], Tabor Scott Mclaughlin[1], Rama Kishore Annavarapu[1], Hossein Sojoudi*[1]

[1]Dept. of Mechanical, Industrial and Manufacturing Engineering, The University of Toledo

*Corresponding author. email: hossein.sojoudi@utoledo.edu

Reasoning for Model

[We]: $We = \rho v^2 R_0/\sigma$

[Re]: $Re = \rho v R_0/\mu$

[1] $\xi_{\max} = \frac{2}{3}\sqrt{\frac{2\left(We+6\right)}{\frac{We}{\sqrt{2Re}}+1-\cos\theta_{\text{eq}}}}\cos\alpha \mid \alpha = \frac{1}{3}\arccos\left(18\sqrt{\frac{\frac{We}{\sqrt{2Re}}+1-\cos\theta_{eq}}{2\left(We+6\right)^3}}\right)$

(From **Zhao *et al.*,** model for maximum spreading)

[2]: $\frac{4\pi}{3} * R_0^3 = \pi * R_{\max}^2 * h \Rightarrow \frac{4R_0}{3h} = \frac{R_{\max}^2}{R_0^2} = \xi_{\max}^2$ (**conservation of mass**)

[3]: Empirical relation from Experimentation, $a_{\text{effective}} \approx \frac{7}{5} a_{\text{actual}}$

[4]: For droplet to split, $h < a_{\text{effective}}$ (**Bird *et al.***)

$$\Rightarrow h_{\text{critical}} = a_{\text{effective}} = \frac{7}{5} a_{\text{actual}} = \frac{7}{5} a$$

[5]: From [2] & [4]: $\frac{4R_0}{3*\frac{7}{5}a} = \frac{20R_0}{21a} = \xi_{\max}^2$

[6]: From [1]: $\xi_{\max}^2 = \frac{8*\left(We+6\right)}{9\left(\frac{We}{\sqrt{2Re}}+1-\cos\theta_{eq}\right)} * \left(\cos\alpha\right)^2$

[7]: From [5] & [6]: $\frac{15R_0}{14a} = \frac{\left(We+6\right)}{\frac{We}{\sqrt{2Re}}+1-\cos\theta_{eq}} * \left(\cos\alpha\right)^2$

[8] From [7], [We], & [Re]: $\frac{15R_0}{14a} = \frac{\left(\frac{\rho v^2 R_0}{\sigma}+6\right)}{\frac{\sqrt{\rho R_0 \mu}}{\sqrt{2}*\sigma} * v^{\frac{3}{2}} + 1 - \cos\theta_{eq}} * \left(\cos\alpha\right)^2$

$$\Rightarrow \frac{\rho R_0 \cos^2\alpha}{\sigma} v^2 - \frac{15R_0\sqrt{\rho R_0 \mu}}{14\sqrt{2}a\sigma} v^{\frac{3}{2}} - \frac{15R_0}{14a}\left(1-\cos\theta_{\text{eq}}\right) + 6\cos^2\alpha = 0$$

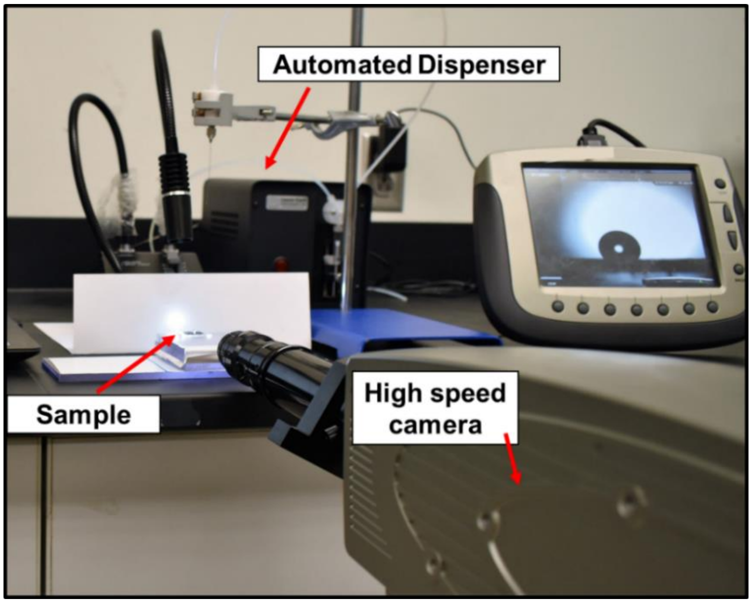

Supplementary Figure 1 | Experimental setup. The sample was placed on a horizontal stage, above which liquid droplets are generated by pumping liquids through a steel needle using a ramé-hart Automated Dispensing System. Drop Volume Control software was used to control the liquid input-output and for purging the whole dispensing channel. Impact velocity (*v*) was varied by adjusting the releasing height. A high-speed video camera was used to capture slow motion droplet impacts typically at 10,000 fps.

Table S1 Calculated energies of impacting droplets with various glycerol-water mixture (0%, 40%, 60% and 70% by weight) liquids at two different impact velocities based on the energy equations mentioned in the main article.

Impact velocity, v (m/s)	Glycerol-water weight (%)	Energy (J)			
		Kinetic, KE_1	Surface, SE_1	Surface, SE_2	Dissipation, E_{diss}
1	0	4.96031E-06	1.53E-06	7.41E-07	2.51E-07
	40	5.40673E-06	1.4E-06	6.74E-07	9.39E-07
	60	5.70435E-06	1.37E-06	6.54E-07	2.4E-06
	70	5.85316E-06	1.35E-06	6.35E-07	3.74E-06
2	0	1.84056E-05	1.53E-06	7.41E-07	4.5E-06
	40	2.00621E-05	1.4E-06	6.74E-07	1.54E-05
	60	2.11664E-05	1.37E-06	6.54E-07	3.55E-05
	70	2.17186E-05	1.35E-06	6.35E-07	4.214E-05

Table S2 Comparison between theoretical and experimental values of minimum impact velocities for splitting (v_{MS}) with various glycerol-water mixture (0%, 40%, 60% and 70% by weight) liquid droplets of different volumes (9.2 µL, 7.2 µL and 5.5 µL). Macrotexture size, a = 180 µm.

Glycerol-water weight (%)	Droplet volume (µL)	Minimum impact velocity for splitting, v_{MS} (m/s)	
		Theoretical	Experimental
0	9.2	0.99	0.93 ± 0.03
	7.2	0.95	0.90 ± 0.03
	5.5	0.93	0.86 ± 0.03
40	9.2	1.02	0.96 ± 0.03
	7.2	0.98	0.90 ± 0.03
	5.5	0.95	0.88 ± 0.03
60	9.2	1.20	1.19 ± 0.02
	7.2	1.15	1.16 ± 0.02
	5.5	1.11	1.12 ± 0.02
70	9.2	1.51	1.46 ± 0.02
	7.2	1.43	1.39 ± 0.02
	5.5	1.37	1.30 ± 0.02

Additional Supplementary Files

Supplementary Movie 1: The eight different scenarios of the 9.2 μL droplets impacting superamphiphobic surfaces at a low impact velocity, v = 1 m/s (We = 18 ~ 24). The top four videos represent the liquid droplets of 0%, 40%, 60%, and 70% glycerol-water mixtures by weight impacting the surfaces without any macrotexture. The bottom four videos represent the corresponding liquid droplets impacting surfaces with the macrotexture.

Supplementary Movie 2: The movie shows eight different scenarios of the 9.2 μL droplets impacting superamphiphobic surfaces at a high impact velocity, v = 2 m/s (We = 71 ~ 94). The top four videos represent the liquid droplets of 0%, 40%, 60%, and 70% glycerol-water mixtures by weight which impacts the surfaces without any macrotexture. The bottom four videos represent the corresponding liquid droplets impacting surfaces with the macrotexture.

Supplementary Movie 3: Comparison of various glycerol-water mixture (0%, 40%, 60%, and 70% by weight) droplets impacting surfaces with and without the macrotexture at their critical impact velocities.
